# Identification of four hub genes as promising biomarkers to evaluate the prognosis of ovarian cancer in silico

**DOI:** 10.1186/s12935-020-01361-1

**Published:** 2020-06-24

**Authors:** Jingxuan Chen, Yun Cai, Rui Xu, Jiadong Pan, Jie Zhou, Jie Mei

**Affiliations:** 1 School of Basic Medical Sciences, Nanjing Medical University, Nanjing, 211166 China; 2 Cytoskeleton Research Group & First Clinical Medicine College, Nanjing Medical University, No. 101 Longmian Road, Nanjing, 211166 China; 3 Department of Bioinformatics, Nanjing Medical University, Nanjing, 211166 China; 4 First Clinical Medicine College, Nanjing Medical University, Nanjing, 211166 China; 5 Department of Gynecology and Obstetrics, Affiliated Wuxi Maternal and Child Health Hospital of Nanjing Medical University, No.48, Huaishu Road, Wuxi, 214023 China

**Keywords:** Ovarian cancer, WGCNA, Bioinformatic analysis, Prognosis

## Abstract

**Background:**

Ovarian cancer (OvCa) is one of the most fatal cancers among females in the world. With growing numbers of individuals diagnosed with OvCa ending in deaths, it is urgent to further explore the potential mechanisms of OvCa oncogenesis and development and related biomarkers.

**Methods:**

The gene expression profiles of GSE49997 were downloaded from the Gene Expression Omnibus (GEO) database. Weighted gene co-expression network analysis (WGCNA) was applied to explore the most potent gene modules associated with the overall survival (OS) and progression-free survival (PFS) events of OvCa patients, and the prognostic values of these genes were exhibited and validated based on data from training and validation sets. Next, protein–protein interaction (PPI) networks were built by GeneMANIA. Besides, enrichment analysis was conducted using DAVID website.

**Results:**

According to the WGCNA analysis, a total of eight modules were identified and four hub genes (MM > 0.90) in the blue module were reserved for next analysis. Kaplan–Meier analysis exhibited that these four hub genes were significantly associated with worse OS and PFS in the patient cohort from GSE49997. Moreover, we validated the short-term (4-years) and long-term prognostic values based on the GSE9891 data, respectively. Last, PPI networks analysis, Gene Ontology (GO) and Kyoto Encyclopedia of Genes and Genomes (KEGG) analysis revealed several potential mechanisms of four hub genes and their co-operators participating in OvCa progression.

**Conclusion:**

Four hub genes (*COL6A3*, *CRISPLD2*, *FBN1* and *SERPINF1*) were identified to be associated with the prognosis in OvCa, which might be used as monitoring biomarkers to evaluate survival time of OvCa patients.

## Background

Ovarian cancer (OvCa) is a common cancer which has the highest morbidity and quietly poor prognosis among gynecological malignancies worldwide. In United States, OvCa causes approximately 14 thousands death patients in 2018 [1]. With the continuous improvement of comprehensive therapy, patients with early stage OvCa seem to have satisfactory prognosis that the 5-year survival rate reaching 93%. Nevertheless, since the majority of patients, precisely more than 80%, would be hard to be diagnosed until the tumor at FIGO stage III or stage IV, leading to a considerable number of mortality [2]. Hence, increasing number of researchers focus on this horrible disease and attempt to explore novel procedures for early diagnosis and treatment. However, early diagnostic strategies and reliable models to guide therapy and evaluate prognosis have been lacking up to now.

In the past decades, vigorously developing computer technology has largely promoted the flourish of big data applications. As an emerging biomedical auxiliary research technology, bioinformatics analysis has been widely applied to several aspects of clinical or basic medical research. Weighted gene co-expression network analysis (WGCNA) is a systematic biological method which describes the pattern of gene association between different samples [3]. Researchers utilize WGCNA to identify gene sets of interest with information of thousands of significantly altered genes or all genes, and then perform significant association analyses with phenotypes. At present, several laboratories have applied this technology into their researches [4–6]. Meanwhile, in the field of cancer research, investigators tend to take advantage of WGCNA for systematic analysis of phenotypes, especially for developing novel prognostic models [7, 8].

GSE49997, a microarray containing 204 OvCa samples, was contributed by Dietmar et al. in 2014 [9]. Dietmar et al. validated the prognostic impacts of a molecular subtype in OvCa on overall survival (OS) and progression-free survival (PFS) based on this microarray [10]. Given the integrated follow-up information and gene expression data in this microarray, we re-assessed the above-mentioned data and finally appraised four potential biomarkers predicting prognosis of OvCa patients through WGCNA analysis. We further conducted verification analysis on prognostic values of four genes through another microarray, GSE9891. In conclusion, we found out four genes (*COL6A3*, *CRISPLD2*, *FBN1* and *SERPINF1*) associated poor prognosis, suggesting these genes function as potential biomarkers to evaluate the prognosis of OvCa patients.

## Materials and methods

### Acquisition of microarray data and pre-process

**Fig. 1:**
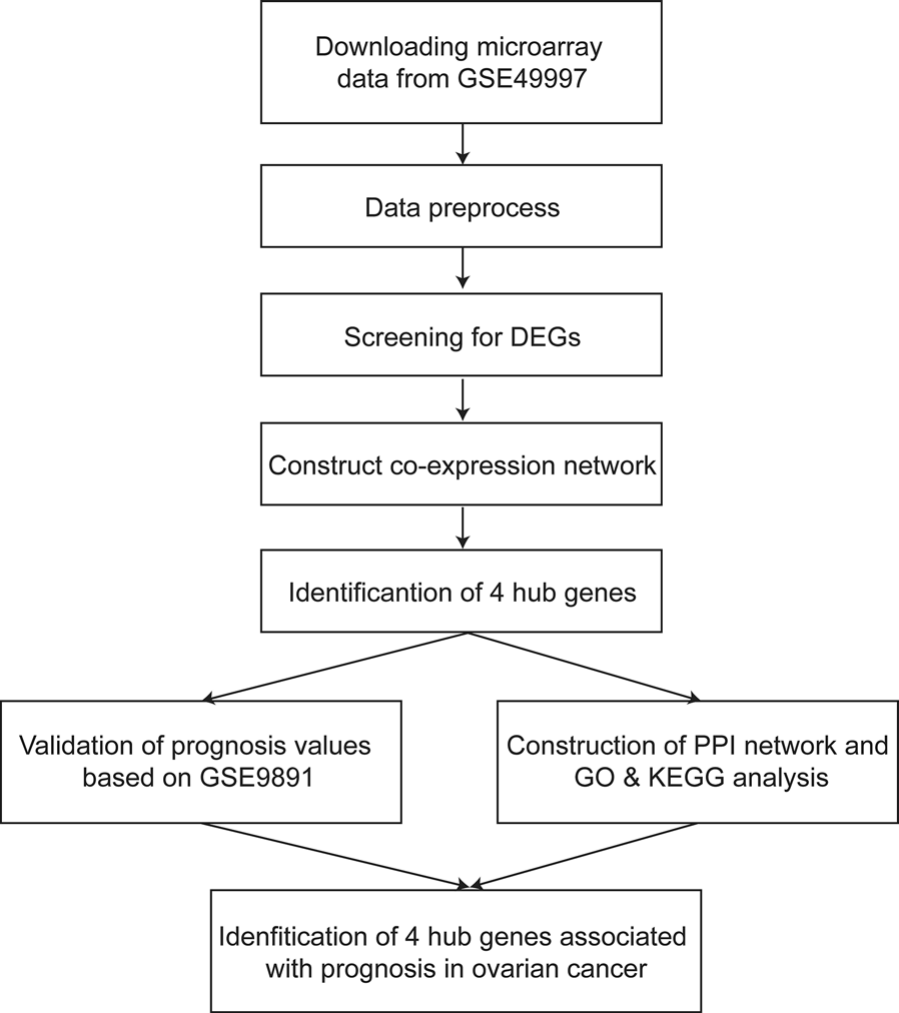
Flow chart of the research. The gene expression profiles of GSE49997 were downloaded from the GEO database. WGCNA was applied to investigate potential biomarkers associated with the OS and PFS events. Besides, the short-term and long-term prognostic value of hub genes was validated based on data from GSE9891. In addition, the PPI networks were constructed by GeneMANIA and enrichment analysis was further conducted to reveal the potential mechanisms of four hub genes and their cooperator participating in OvCa progression

The workflow of our research was summarized in Fig. 1. The array profiles of GSE49997 (https://www.ncbi.nlm.nih.gov/geo/query/acc.cgi?acc=GSE49997) [9] were downloaded from the Gene Expression Omnibus (GEO) database. Besides, four hub gene expression data and survival data corresponding to GSE9891 were downloaded from the Kaplan–Meier Plotter (http://kmplot.com/analysis/) [11]. The basic clinic-pathological features of two microarrays were described in Table 1 according to original publications of GSE49997 and GSE9891 [9, 10]. Before further analysis, array profiles of GSE49997 were pre-processed by background correction, quantile normalization and probe summarization. After matching the gene expression data and survival information, 194 sample from GSE49997 were retained in the current research. For further WGCNA analysis, the top 25% different expression genes (DEGs) from GSE49997 dataset according to analysis of variance (3,837 genes) were retained.

**Table 1:** The basic clinic-pathological features of OvCa patients in two datasets

Characteristics	n	Cases (%)	
		GSE49997	GSE9891
Histologic subtype			
Serous	435	171 (88.1%)	264 (92.6%)
Non-serous	44	23 (11.9%)	21 (7.4%)
FIGO stage			
Stage 1	24	0 (0%)	24 (8.4%)
Stage 2	27	9 (4.6%)	18 (6.3%)
Stage 3	371	154 (79.4%)	217 (76.1%)
Stage 4	53	31 (16.0%)	22 (7.7%)
Unknown	4	0 (0%)	4 (1.4%)
Grade			
Grade 1	30	11 (5.7%)	19 (6.7%)
Grade 2	136	39 (20.1%)	97 (34.0%)
Grade 3	307	143 (73.7%)	164 (57.5%)
Unknown	6	1 (0.5%)	5 (1.8%)
Residual tumor			
No	221	137 (70.6%)	84 (29.5%)
Yes	221	57 (29.4%)	164 (57.5%)
Unknown	37	0 (0%)	37 (13.0%)

### Co-expression network construction and identification of hub genes

After pre-processing the GSE49997 microarray data, the expression profile of these 3,837 genes was sent to construct a gene co-expression network using the WGCNA package in R language [12]. The idea of a soft threshold is to continually elementize the elements in the Adjacency Matrix through a weight function and the choice of the soft threshold β is bound to affect the result of module identification. To create a network with a nearly scale-free topology, we installed the soft threshold power of β = 3 (scale free R^2^ = 0.868). Adjacency matrices were calculated and transformed into the topological overlap matrix (TOM). The dynamic tree cut algorithm was applied to detect gene modules. Gene significance (GS) was defined as the correlation coefficient between gene expression and module traits. The module eigengene was calculated as a summary profile for each module. Module significance was defined as the correlation coefficient between a module’s eigengene and traits. Module membership (MM) was defined by the correlation coefficient of the module eigengene and gene expression profile. Genes with MM values above 0.90 were considered to be the modules’ representative genes with potential critical functions.

### Survival analyses and further authentication of key genes

The prognostic impacts of four genes for OS and PFS were evaluated by Kaplan–Meier analysis. To further verify the significant prognostic values of four hub genes, we used the array profiles and clinical data from GSE9891 to conduct survival analysis. Given that the follow-up information from training set was collected within about 4 year, we firstly set 48 months as the end point of follow-up to evaluate the short-term prognostic values of four genes. Besides, the long-term prognostic values were also validated by taking full advantage of the survival data from the validation set. For Kaplan–Meier analysis, all cases were ranked based on hub genes expression levels and further divided into two groups according to the median expression of these genes.

### Construction of protein–protein interaction network

Protein–protein interaction (PPI) network was been constructed by GeneMANIA (https://genemania.org/) [13], an online server that explore interconnections between proteins in term of physical interaction, co-expression, predicted, co-localization, common pathway, genetic interaction and shared protein domains. In this research, GeneMANIA was used for PPI analysis of four hub genes at the gene level.

### Gene Ontology and Kyoto Encyclopedia of Genes and Genomes analysis

The Database for Annotation, Visualization and Integrated Discovery (DAVID, https://david.ncifcrf.gov/) [14] was applied to perform Gene Ontology (GO) and Kyoto Encyclopedia of Genes and Genomes (KEGG) analyses of four hub genes and their most relevant cooperators. The human genome (Homo sapiens) was selected as the background variables. Enrichment terms were considered statistically significant when the FDR were less than 0.05 and the top 10 terms of each analysis were retained to plot bubble chart.

### Statistical analysis

All statistical analyses were performed using SPSS 25.0 software and R 3.5.1 software. Kaplan–Meier survival plots were generated with survival curves compared by log-rank test, but Tarone-Ware test was used when obvious crossover between the groups was observed in survival plots. For all analyses, differences were considered statistically significant if the P values were less than 0.05.

## Results

### Weighted co-expression network construction and key hub genes identification

**Fig. 2:**
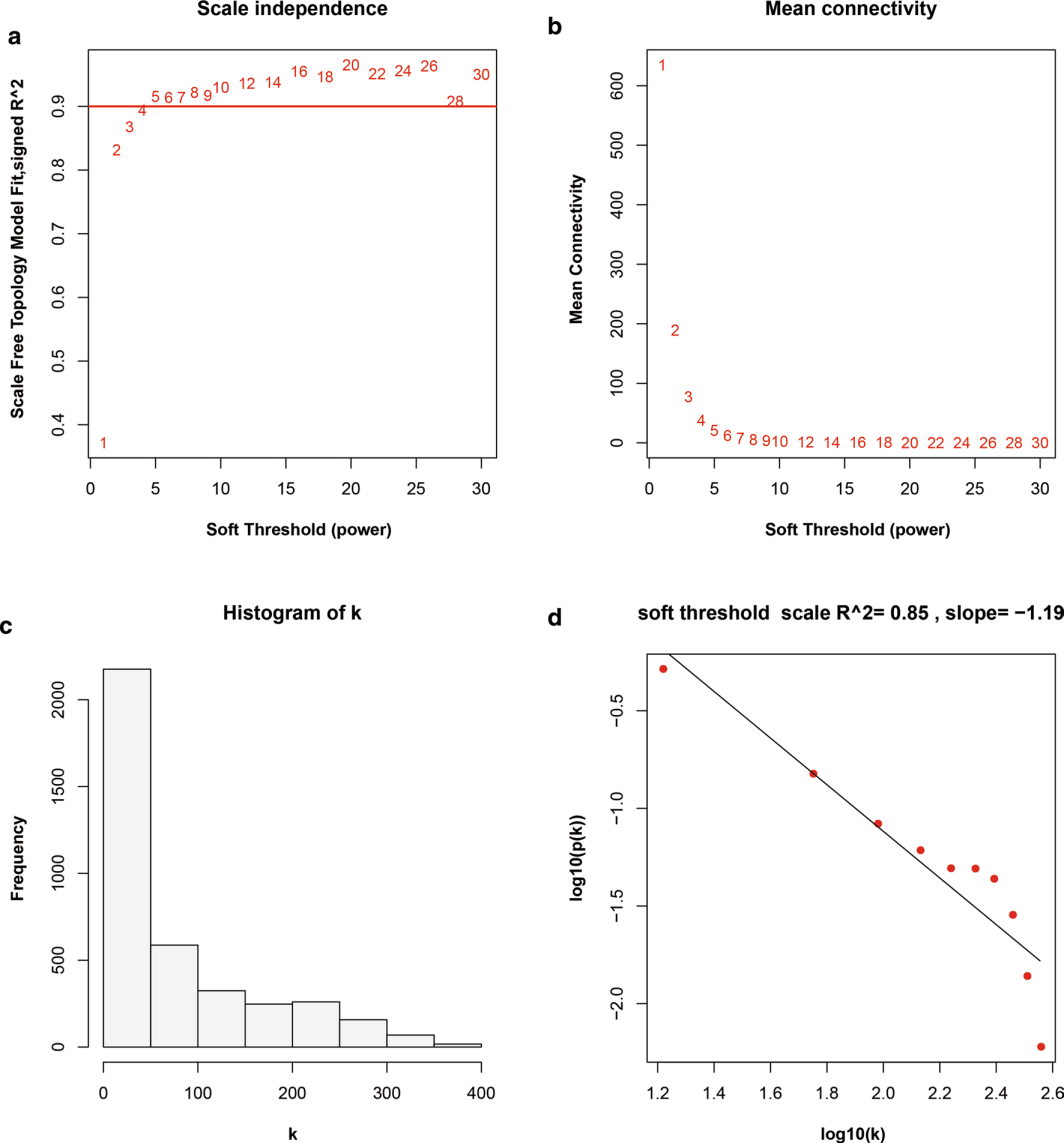
Determination of soft-thresholding power in WGCNA. **a** Analysis of the scale-free fitting indices for various soft-thresholding powers (β). **b** Mean connectivity analysis of various soft-thresholding powers. **c** Histogram of the connection distribution when β = 3. **d** Checking the scale-free topology when β = 3. According to **c**, **d**, k and p(k) were negatively correlated (correlation coefficient is 0.85), indicating that a gene scale-free network can be resumed

**Fig. 3:**
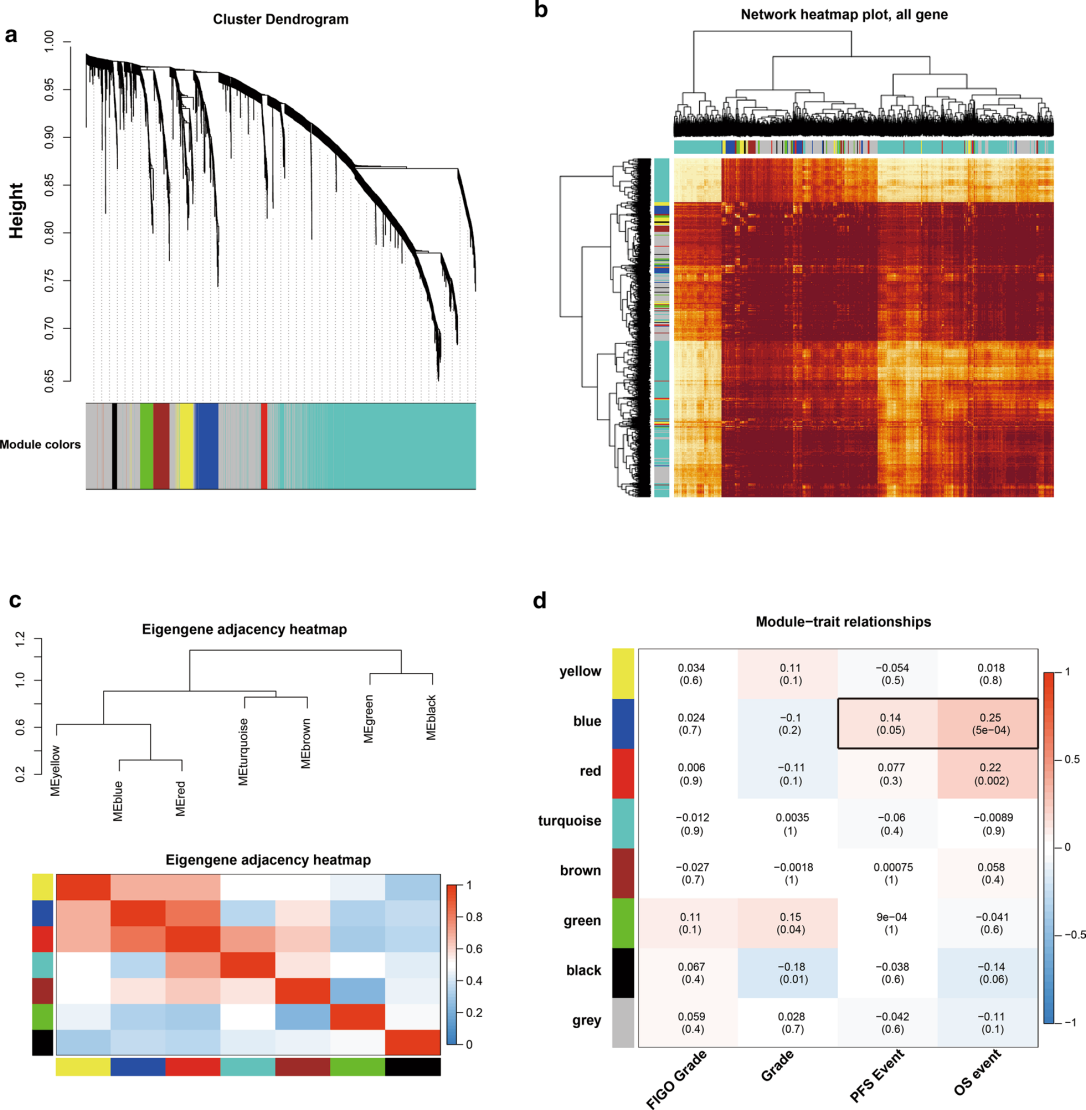
Identification of relevant modules associated with OvCa clinical traits. **a** Clustering dendrograms of genes were based on dissimilarity topological overlap and module colours. As a result, 8 co-expression modules were constructed and are shown in different colours. These modules were arranged from large to small according to the number of genes included. **b** This heatmap depicted the TOM among all genes in the analysis. A light colour represented low overlap, and progressively darker reds indicated higher overlap. Blocks of darker colours along the diagonal were the modules. The gene dendrogram and module assignment were also shown along the left and upper sides. **c** The eigengene dendrogram and heatmap identify groups of correlated with eigengenes termed meta-modules. As a result, the dendrogram showed that blue modules were highly related to OvCa patients’ survival. **d** Heatmap of the correlation between module eigengenes and clinical traits of OvCa. The blue gene module was revealed to exhibit the highest correlation with both OS and PFS events

We applied the R package for WGCNA in the construction of a co-expression network and then 3837 DEGs with similar expression features were submitted to modules through cluster analysis. In our research, we selected the power of β = 3 (scale free R^2^ = 0.868) as the soft threshold to ensure a scale-free network (Fig. 2a–d). Then, we extracted eight modules for next analysis (Fig. 3a). Afterwards, we took advantage of a heatmap and meta-modules aiming to visualize the gene network (Fig. 3b, c). The blue module, most remarkably correlated with both OS (R^2^ = 0.25; P = 5e−4) and PFS (R^2^ = 0.14; P = 0.05) events, was shown to be of remarkable value in the evaluation of OvCa prognosis. Subsequently, we selected 4 genes (*COL6A3*, *CRISPLD2*, *FBN1* and *SERPINF1*) in the blue module with MM values above 0.90 for further analysis, which were regarded as typical genes to exhibit crucial functions (Table 2).

**Table 2:** Four hub genes identified by WGCNA analysis in OvCa

Gene ID	Ensembl ID	Gene description
COL6A3	ENSG00000163359.15	Collagen, type VI, alpha 3
CRISPLD2	ENSG00000103196.11	Cysteine-rich secretory protein LCCL domain containing 2
FBN1	ENSG00000166147.13	Fibrillin 1
SERPINF1	ENSG00000132386.10	Serpin peptidase inhibitor, clade F, member 1

### Prognostic value of key genes in predict OS and RFS in ovarian

**Fig. 4:**
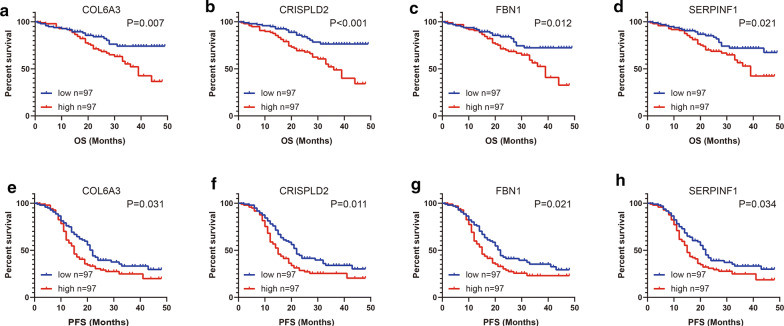
The prognostic values of four genes in training dataset. The patients in GSE49997 were divided into a high-expression group and a low-expression group according to the median gene expression. The prognostic values of **a** COL6A3, **b** CRISPLD2, **c** FBN1 and **d** SERPINF1 expression in predicting OS in OvCa patients. The prognostic values of **e** COL6A3, **f** CRISPLD2, **g** FBN1 and **h** SERPINF1 expression in predicting PFS in OvCa patients

To assess the prognostic values of four hub genes, we next performed Kaplan–Meier analysis. As shown in Fig. 4, high mRNA expression levels of COL6A3 (P = 0.007), CRISPLD2 (P < 0.001), FBN1 (P = 0.012) and SERPINF1 (P = 0.021) were significantly associated with worse OS in OvCa patients. Meanwhile, high mRNA expression levels of COL6A3 (P = 0.031), CRISPLD2 (P = 0.011), FBN1 (P = 0.021) and SERPINF1 (P = 0.034) were notably associated with poor PFS as well. Overall, these findings revealed the promising prognostic values of four hub genes in OvCa.

### Validation of hub genes based on GSE9891 microarray data

**Fig. 5:**
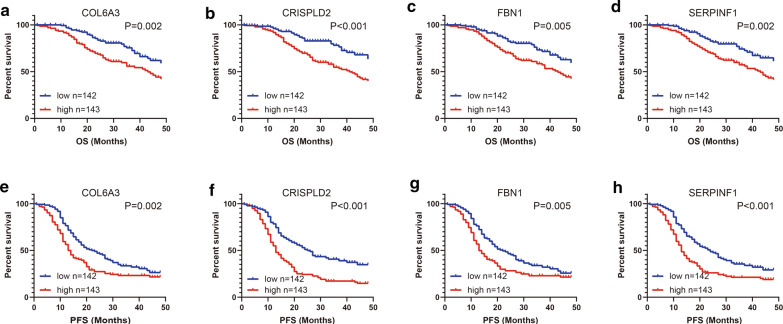
The short-term prognostic values of four genes in validation dataset. The patients in GSE9891 were divided into a high-expression group and a low-expression group according to the median gene expression. Forty-eight months were treated as the end point of follow-up. The prognostic values of **a** COL6A3, **b** CRISPLD2, **c** FBN1 and **d** SERPINF1 expression in predicting OS in OvCa patients. The prognostic values of **e** COL6A3, **f** CRISPLD2, **g** FBN1 and **h** SERPINF1 expression in predicting PFS in OvCa patients

To validate the prognostic values of these four hub genes in OvCa, we used microarray data from GSE9891 to execute survival analysis for the four hub genes. Given that the follow-up information from training dataset (GSE49997) was collected within 4 year, we firstly set 48 months as the end point of follow-up to assess the short-term prognostic values of four genes. At first, to guarantee the homogeneity of two datasets, we compared the difference between OS and PFS events, and the result showed that no statistical significance was found (Table 3). As shown in Fig. 5, high mRNA expression levels of COL6A3 (P = 0.002), CRISPLD2 (P < 0.001), FBN1 (P = 0.005) and SERPINF1 (P = 0.002) were remarkably associated with worse OS in OvCa patients. Meanwhile, high mRNA expression levels of COL6A3 (P = 0.002), CRISPLD2 (P < 0.001), FBN1 (P = 0.005) and SERPINF1 (P < 0.001) were also notably associated with unfavorable PFS.

**Table 3:** Comparison of patents’ survival events in two datasets

Survival events	n	Cases (%)		χ^2^	P value
		GSE49997	GSE9891		
OS event				0.83	> 0.05
Alive	312	137 (70.6%)	190 (66.7%)		
Dead	167	57 (29.4%)	95 (33.3%)		
PFS event				0.15	> 0.05
Non-progressive	162	70 (36.1%)	98 (34.4%)		
Progressive	317	124 (63.9%)	187 (65.6%)

**Fig. 6:**
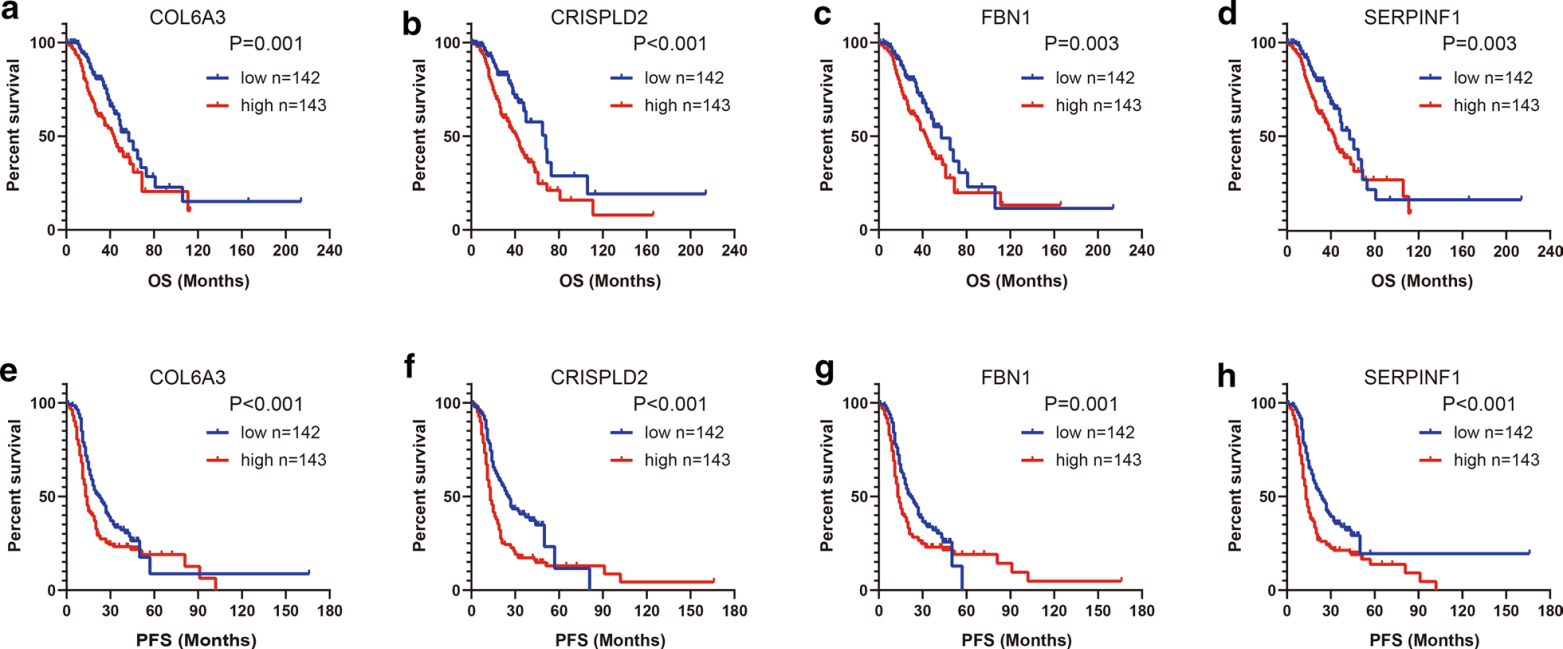
The long-term prognostic values of four genes in validation dataset. The patients in GSE9891 were divided into a high-expression group and a low-expression group according to the median gene expression. The prognostic values of **a** COL6A3, **b** CRISPLD2, **c** FBN1 and **d** SERPINF1 expression in predicting OS in OvCa patients. The prognostic values of **e** COL6A3, **f** CRISPLD2, **g** FBN1 and **h** SERPINF1 expression in predicting PFS in OvCa patients

Furthermore, the long-term prognostic values were also validated by taking full advantage of the survival data from the validation dataset. As shown in Fig. 6, high mRNA expression levels of COL6A3 (P = 0.001), CRISPLD2 (P < 0.001), FBN1 (P = 0.003) and SERPINF1 (P = 0.003) were remarkably associated with worse OS. Besides, high mRNA expression levels of COL6A3 (P < 0.001), CRISPLD2 (P < 0.001), FBN1 (P = 0.001) and SERPINF1 (P < 0.001) were significantly associated with unfavorable PFS in OvCa patients as well. Overall, these findings validated the prognostic values of these four hub genes in OvCa.

### Construction of PPI networks and enrichment analysis

**Fig. 7:**
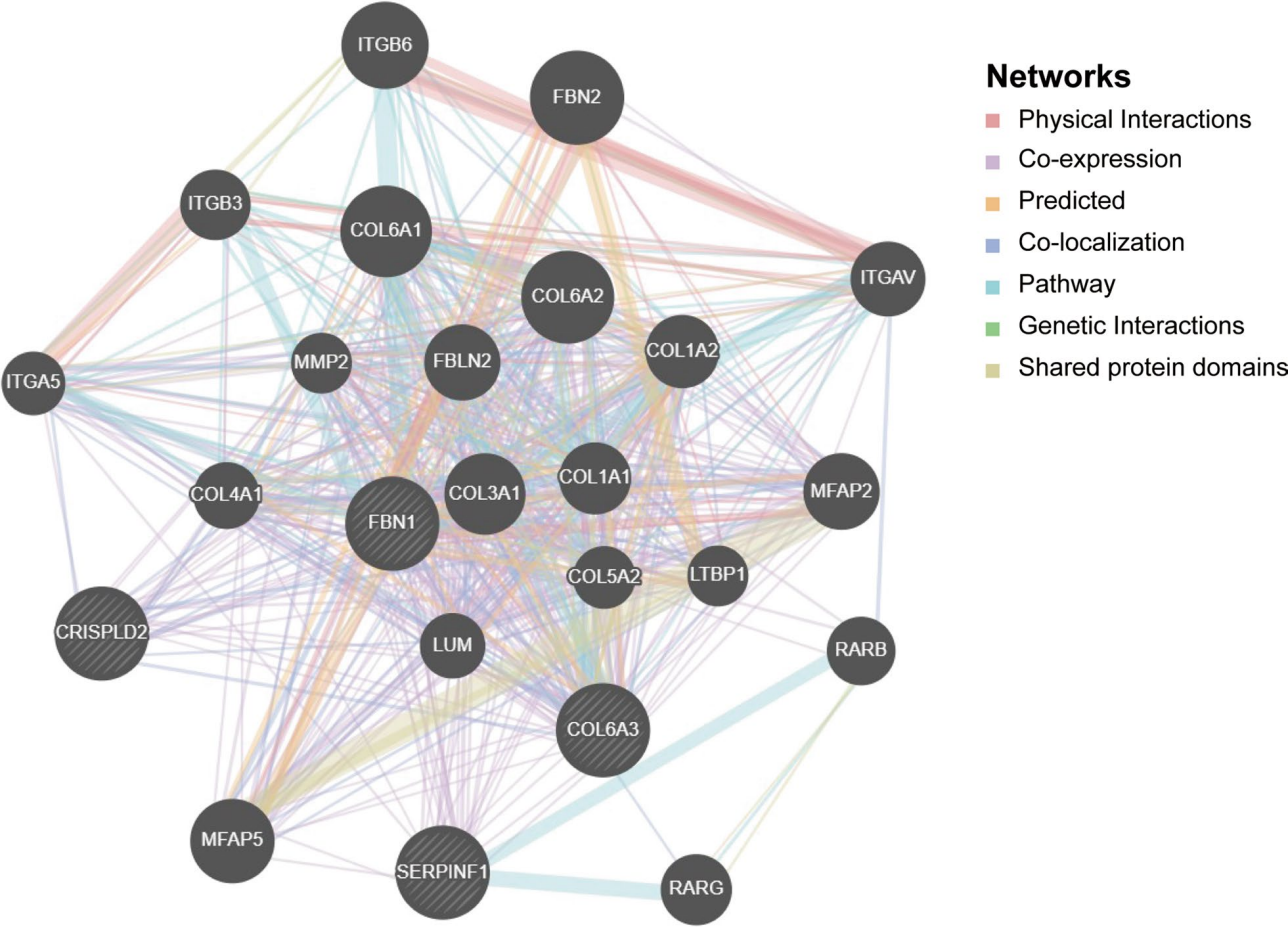
The PPI network of four genes constructed by GeneMANIA. PPI network for four hub genes was constructed in GeneMANIA website. The interconnections between proteins were explored in term of physical interaction, co-expression, predicted, co-localization, common pathway, genetic interaction and shared protein domains

**Fig. 8:**
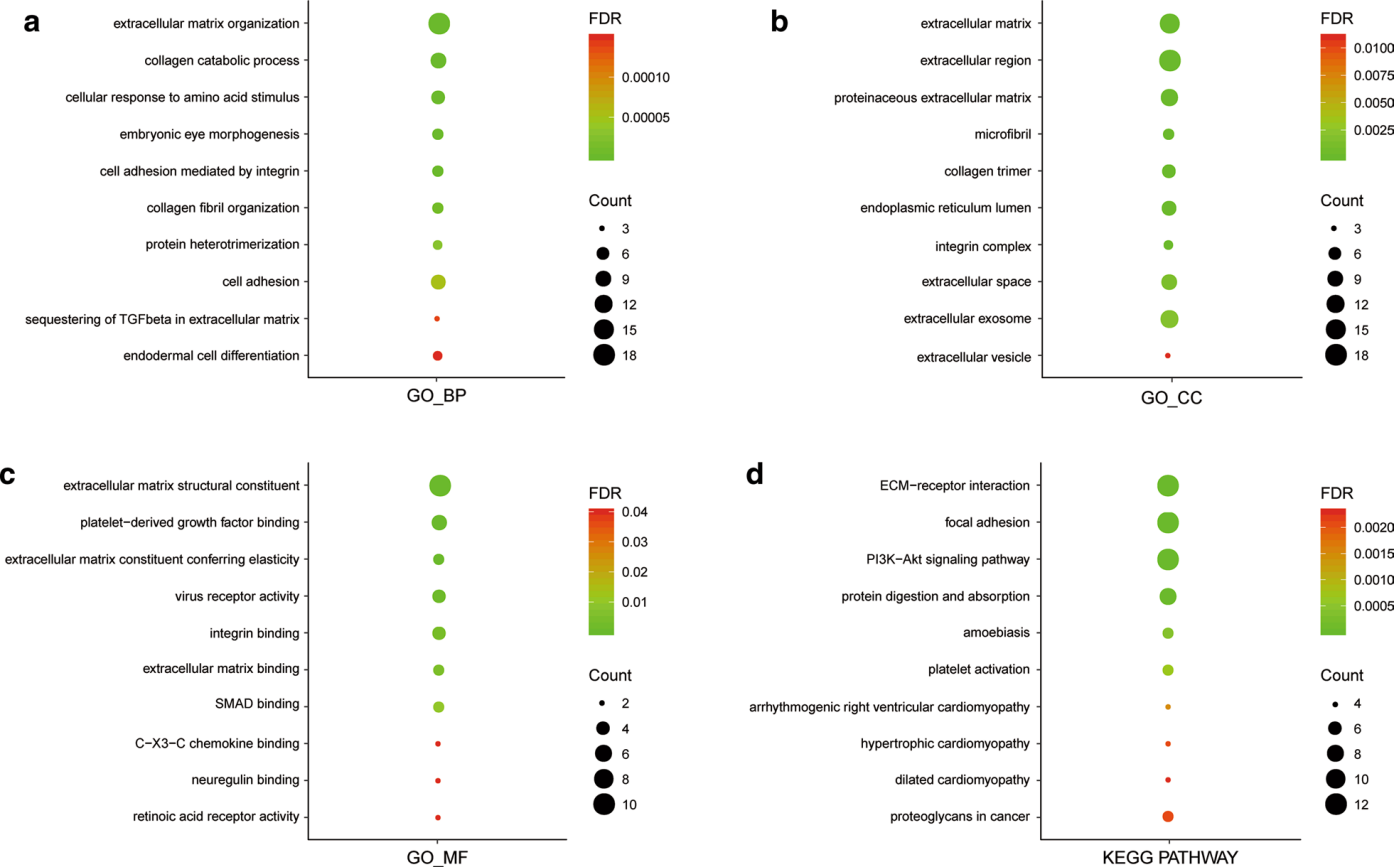
GO and KEGG pathway enrichment analysis. Enrichment analysis was performed to preliminarily explain the potential mechanisms of hub genes and their co-operators. **a** biological process, **b** cellular component, **c** molecular function and **d** KEGG pathway analysis. The size of each circle indicated the counting number on each part, while the color represents the FDR of the enrichment analysis

To explore potential mechanisms that these four hub genes participate in the carcinogenesis of OvCa, we applied GeneMANIA to set up a PPI network for these genes and the results revealed a series of cooperators (Fig. 7). Next, GO and KEGG analyses based on DAVID website were performed to identify the functional enrichment of four hub genes and the interacted genes. GO analysis included three main functions of selected genes, including biological process (BP), cellular components (CC), and molecular functions (MF). The relative findings were represented in Fig. 8. Besides, we supplied all GO and KEGG terms with statistical difference as Additional file 1: Table S1. These results revealed several potential mechanisms of four hub genes and their co-operators participating in OvCa progression, which provide novel insights for further study.

## Discussion

Currently, human genome research has moved towards a central phase. The successful expiry of the Human Genome Project marks a turning point of genomic research, meaning that the human genome research approaching a brand new stage of information extraction and data analysis [15, 16]. Bioinformatics is a new interdisciplinary subject developed in this context that combines the theoretical methods of biology, mathematics, physics, information science, and computer science. The core of bioinformatics is genomic informatics, including the acquisition, processing, storage, distribution and interpretation of genomic information. The key to genomic informatics is to “read” the nucleotide sequence of the genome, precisely, the exact location of all genes on the chromosome and the function of each DNA fragment [17]. Increasing evidence indicates that with the development of bioinformatics, we afford to use data analysis techniques to determine the role of mutations in tumor formation. Identification of molecular biomarkers and expression profiles is frequently adopted in tumor systematization, diagnosis, and prediction of prognosis. The identification of oncogenesis-related genes, proteins, and cellular pathways facillitates the researches for more effective therapeutic drugs [18].

Co-expression analysis is an efficient strategy for gene/disease prediction analysis in large-scale datasets. In this research, we applied WGCNA to construct a gene co-expression network, to evaluate the relationships between genes and modules and to study the relationships between modules and clinical traits. In the analysis of the top 25% most variant genes, the blue module was identified to present the closest correlation with OS and PFS events, and 4 genes (*COL6A3*, *CRISPLD2*, *FBN1* and *SERPINF1*) with high connectivity were filtered from this module.

COL6A3 encodes proteins to be the component of a beaded filament collagen, which can be found in majority of connective tissues. Alpha-3 chain presents larger than other two kinds of chains composing collagen VI, for increasing number of a shared subdomain. As a primary cell-adhesive protein, collagen VI family frames a microfibrillar network supporting the function of skeletal muscle, skin, and cartilage, widely found in extracellular matrices [19, 20]. Mutations in type VI collagen genes easily lead to a range of muscle disorders, from normal defect like Bethlem Myopathy to the severer like Ullrich Scleroatonic Muscular Dystrophy. Recently, researchers have found conclusive evidences suggesting the association between COL6A3 and plurality of cancers [21–23]. COL6A3 is the most upregulated extracellular matrix (ECM) gene in cisplatin-resistant OvCa cells, and cultivation of cisplatin-sensitive cells in the presence of type VI collagen protein promotes resistance in vitro [22]. Besides, COL6A3 is also revealed to be associated with oxaliplatin resistance in OvCa [24].

CRISPLD2 was first identified in rat by Kaplan et al. [25, 26]. There is a potential mechanism that mediating CRISP and LCCL domain containing protein 2 to function in branching morphogenesis, with its nature of a glucocorticoid-inducible gene and part of a cytoskeletal network [27]. Broadly expressing in gall bladder and placenta, LCCL domain variants affect diversity of adult human height [28]. CRISPLD2 has been proved to play significant role in either maintaining cell structure, participating in immune response, inhibiting inflammatory or involving in cell motility [29–32]. Actually, this gene is also implicated in cancers, lung defects, and epithelium growth [32, 33]. However, the role of CRISPLD2 in OvCa has not been observed yet.

As an extracellular matrix glycoprotein, FBN1 has been reported to promote the structure formation of calcium-binding microfibrils. Marfan syndrome seems to show great associations with mutations in *FBN1*, for the reason that the proteins encoded by this gene belongs to a member of the fibrillin family and can be hydrolyzed into FBN1 and hormone Asprosin protein [34, 35]. The former is a type of extracellular matrix component that serves as force-bearing structure support for connective tissue, whether elastic or nonelastic. The latter originates from white adipose tissue and serves as a regulator of glucose homeostasis [36]. Several lines of evidence indicate that FBN1 down-regulates the growth and sprouting of tumor endothelial cells via promoter histone modifications [37]. In OvCa, FBN1 functions as the downstream of Aurora-A and BRCA2, and promoted tumor metastasis through mediating the p53 and SLUG-associated signals [38].

SERPINF1 has nothing to do with serine protease inhibitory activity though this gene encodes a member of serpin family protein. Alternatively, the secreted protein is a type of strong angiogenesis blocker [39]. Moreover, researchers investigated patients with retinoblastoma and found that pathological cells in their body rely on SERPINF1, with its contribution to neuron differentiation. Mutations of this gene are also detected in osteogenesis imperfecta, type VI [39, 40]. Individuals suffering from type II diabetes have increasing circulating level of PEDF, confirmed a strong association with this gene at genome-wide significance [41, 42]. In cancerous diseases, SERPINF1 functions as a tumor suppressor in cervical cancer, which is downregulated by TXNDC5, resulting in stimulating cell migration, vasculogenic mimicry and angiogenesis [43]. Nwani et al. uncovered that SERPINF1 could maintain tumor suppressive functions in fibroblasts to prevent cancer-associated fibroblasts conversion [44]. However, in our research, SERPINF1 expression was associated with unfavorable prognosis, suggesting an opposite role in OvCa.

In our research, we further validated the prognostic values of 4 genes (*COL6A3*, *CRISPLD2*, *FBN1* and *SERPINF1*) in OvCa patients based on another microarray and the findings confirmed the reliable values for prognostic evaluation. Furthermore, we employed GeneMANIA to construct a PPI network and applied GO and KEGG analyses to identify the functional enrichment of four hub genes and the interacted genes and the results revealed several potential mechanisms of four hub genes and their co-operators in participating in OvCa progression. As results shown, four hub genes and their co-operators mainly participated in ECM regulation. The ECM mediates tissue development and homeostasis, while dys-regulation of ECM dynamics have been thought of as crucial drivers for both tumorigenesis and progression of cancers [45]. Taken together, our findings provided novel insights for hub genes mediating ECM-associated OvCa oncogenesis and development.

## Conclusion

Overall, our results furnished valuable directions for biomarker research on OvCa prognostic evaluation. We obtained crucial prognosis-associated genes based on WGCNA analysis and confirmed the prognostic values using data from GSE9891. Finally, four hub genes (*COL6A3*, *CRISPLD2*, *FBN1* and *SERPINF1*) were identified for further research, which might be employed as prospective biomarkers to assess OS and PFS in OvCa patients.

## Supplementary information

**Additional file 1: Table S1.** GO and KEGG pathway enrichment analysis (all terms with statistical difference).

## Data Availability

All data are included in the article.

## Abbreviations

OvCa: Ovarian cancer
WGCNA: Weighted gene co-expression network analysis
OS: Overall survival
PFS: Progression-free survival
GEO: The Gene Expression Omnibus
DEGs: Different expression genes
TOM: Topological overlap matrix
GS: Gene significance
MM: Module membership
PPI: Protein–protein interaction
DAVID: The Database for Annotation, Visualization and Integrated Discovery
GO: Gene Ontology
KEGG: Kyoto Encyclopedia of Genes and Genomes
COL6A3: Collagen, type VI, alpha 3
CRISPLD2: Cysteine-rich secretory protein LCCL domain containing 2
FBN1: Fibrillin 1
SERPINF1: Serpin peptidase inhibitor, clade F, member 1
BP: Biological process
CC: Cellular components
MF: Molecular functions
ECM: Extracellular matrix
